# Did medical doctors who order abdominal CT scans during on-call hours truly become worse at clinical reasoning? Yes, they did

**DOI:** 10.1007/s00330-022-09121-7

**Published:** 2022-09-07

**Authors:** Selin Ersoydan, Derya Yakar, Ömer Kasalak, Thomas C. Kwee

**Affiliations:** Department of Radiology, University Medical Center Groningen, University of Groningen, Groningen, the Netherlands

**Keywords:** Abdomen, Clinical reasoning, Differential diagnosis, Computed tomography, Night shift work

## Abstract

**Objective:**

To investigate temporal changes in clinical reasoning quality of physicians who requested abdominal CT scans at a tertiary care center during on-call hours within a 15-year period.

**Methods:**

This retrospective study included 531 patients who underwent abdominal CT at a tertiary care center during on-call hours on 36 randomly sampled unique calendar days in each of the years between 2005 and 2019. Clinical reasoning quality was expressed as a percentage (0–100%), taking into account the degree by which the differential diagnoses on the CT request form matched the CT diagnosis. Temporal changes in the quality of clinical reasoning and number of CT scans were assessed using Mann-Kendall tests. Associations between the quality of clinical reasoning with patient age and gender, requesting department, and time of CT scanning were determined with linear regression analyses.

**Results:**

The median annual clinical reasoning score was 14.7% (interquartile range: 12.2 to 16.0%; range: 7.7 to 34.6%). The quality of clinical reasoning significantly decreased between 2005 and 2019 (Mann-Kendall Tau of −0.390, *p* = 0.048), while the number of abdominal CT scans significantly increased (Mann-Kendall tau of 0.790, *p* < 0.001).

**Conclusion:**

The clinical reasoning quality of physicians who request abdominal CT scans during on-call hours has deteriorated over time. Clinical reasoning appears to be worse in younger patients.

**Key Points:**

*• In patients with suspected acute abdominal pathology who are scheduled to undergo CT scanning, referring physicians generally have difficulties in making an accurate pretest (differential) diagnosis.*

*• Clinical reasoning quality of physicians who request acute abdominal CT scans has deteriorated over the years, while the number of CT scans has shown a significant increase.*

*• Clinical reasoning quality appears to be worse in younger patients in this setting.*

## Introduction

Medical imaging utilization keeps on increasing in the Western world, and this particularly applies to CT examinations [1, 2]. This growth in CT examinations is due to the relatively widespread availability of CT systems, continuous improvements in technology (which increase image quality, reduce scan time, and decrease radiation dose), decreasing costs of CT scans, and new clinical CT applications.

Although medical imaging is generally considered beneficial to patients, a part of its increase has been attributed to overutilization [3]. Anecdotal data indicate that clinicians request more and more imaging simply because it is available, and to rule out disease rather than to confirm disease that is most likely based on probabilistic hypothetico-deductive reasoning [4–6]. Based on these anecdotal data and our own experience, it is hypothesized that the clinical reasoning quality of physicians who request medical imaging has deteriorated over the years. However, establishing a good differential diagnosis based on clinical history, physical examination, and laboratory findings, is considered important to optimize the interpretation and diagnostic yield of an imaging examination [4–7]. In addition, a good differential diagnosis is necessary to avoid unnecessary imaging studies that may be costly, that may have potential side effects due to the use of ionizing radiation and contrast agents, and that may yield incidental findings.

During on-call hours, CT scans of the abdomen are frequently ordered, because the list of acute abdominal diseases is long and they may be challenging to diagnose based on clinical grounds [8]. As such, they provide an excellent setting to test our hypothesis. The purpose of our study was therefore to investigate temporal changes in clinical reasoning quality of physicians who requested abdominal CT scans at a tertiary care center during on-call hours within a 15-year period.

## Materials and methods

### Study design

This retrospective study was approved by the institutional review board and the requirement for informed consent was waived. A random sample of 36 unique calendar days was selected using the Random Calendar Date Generator [9]. All patients who underwent abdominal CT at a tertiary care center in The Netherlands during on-call evening and night hours on each of these 36 unique calendar days in each of the years between 2005 and 2019, were potentially eligible for inclusion. The University Medical Center Groningen is one of the seven university medical centers in the Netherlands, with more than 2 million people in its direct catchment area in the north-east of the country. It has more than 10,000 employees and 1,300 beds, and all medical specialties are represented. The hospital is also a recognized Level 1 trauma center, and performs all organ transplants. Patients were included if CT scanning was done to evaluate for acute abdominal pathology, and if the scan range included the region from the diaphragm to the pubic symphysis. Patients who underwent CT scanning to guide an intervention (e.g., drainage), patients whose CT scan was performed for non-acute reasons, and patients whose CT report or clinical records were missing, were excluded. Note that the cases that were selected for the present study were derived from a previously published study that had a different purpose, namely to investigate temporal changes in the utilization and patient impact of abdominal CT during duty shifts in terms of negative scan proportion, same-day hospital discharge, and incidental findings [10]. When ordering abdominal CT during on-call hours, the referring physician both calls the attending radiology resident or radiologist, and submits a digital request form. Other details regarding on-call logistics, CT acquisition, and CT interpretation can be read elsewhere [10].

### Data extraction

Patient age, patient gender, indication for CT scanning, requesting department, use of intravenous CT contrast agents, use of gastrointestinal CT contrast agents, time of CT scanning (before or after midnight), and year in which the CT scan was performed, were recorded. The digital request form of each CT scan was evaluated to extract the differential diagnosis that was compiled by the referring physician, and the CT report was evaluated to extract the diagnosis made.

### Data analysis

The quality of referring physicians’ clinical reasoning before ordering a CT scan was quantified using a scoring system. To that end, the differential diagnosis made by the referring physician on the CT request form was compared to the CT diagnosis. Each of the differential diagnoses on the CT request form that matched the CT diagnosis received 1 point. If no differential diagnosis was provided, or if the CT scan diagnosis did not match any of the differential diagnoses on the CT request form, 0 points were assigned. The clinical reasoning score was then calculated by dividing the total number of points by the total number of differential diagnoses that was listed on the CT request form, and expressed as a percentage. For example, if the clinical differential diagnosis included ruptured abdominal aortic aneurysm, urolithiasis, and cholecystitis, and CT demonstrated urolithiasis, the clinical reasoning score would be 33%. Mann-Kendall tests were performed to test for any temporal changes in both clinical reasoning scores in each year between 2005 and 2019, and number of abdominal CT scans during on-call hours on the 36 randomly sampled unique calendar days in each year between 2005 and 2019. Subsequently, linear regression analyses were performed to determine the association between the quality of clinical reasoning (all years combined) and the following variables: patient age, patient gender, requesting department (surgery, internal medicine, emergency medicine, intensive care, or other), and time of CT scanning (before or after midnight). *p*-values < 0.05 were considered statistically significant. Statistical analyses were executed using R version 3.6.3 software (R Foundation for Statistical Computing) and IBM Statistical Package for the Social Sciences (SPSS) version 23 (SPSS).

## Results

### Patients

**Fig. 1:**
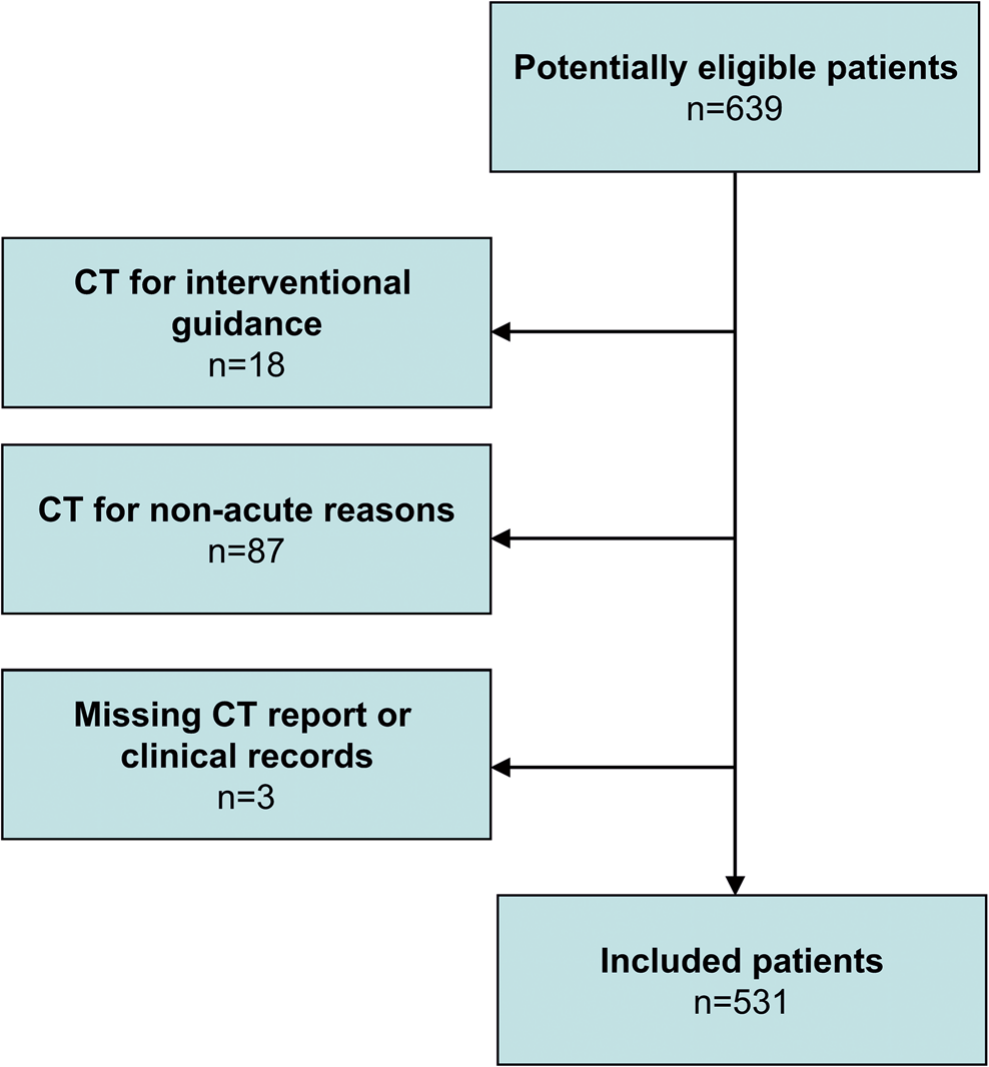
Flowchart of patient selection

The 36 randomly selected unique calendar days are displayed in Table 1. Of 639 potentially eligible patients, 531 remained for inclusion in this study (Fig. 1). In these 531 patients, median age was 59.0 years (interquartile range (IQR): 42.3–70.0 years; range: 10–107 years), male/female ratio was 327/204, requesting department was most frequently surgery (51.8%), indication for CT scanning was most frequently other/mixed (26.6%), most CT scans were performed with intravenous contrast agents in either multiple phases (37.7%) or in the venous phase (37.1%), and without gastrointestinal contrast agents (63.8%), and most CT scans were performed before midnight (75.5%%) (Table 2).

**Table 1:** Thirty-six randomly selected unique calendar days

Month	Day(s)
January	6, 11, 20, 29
February	1, 10, 18
March	1, 4, 21
May	8, 21
June	2, 4, 6, 7, 8, 23, 26, 30
July	7, 14
August	26, 28
September	3, 15, 30
October	2, 9, 11, 21
November	29
December	16, 21, 22, 28

**Table 2:** Characteristics of included cases

Variable		Count (percentage)
Patient age (years)		59.0 (42.3–70.0)^a^
Patient gender	Male	327 (61.6%)
	Female	204 (38.4%)
Requesting department	Surgery	275 (51.8%)
	Internal medicine	82 (15.4%)
	Emergency medicine	67 (29.0%)
	Intensive care	21 (4.0%)
	Other	86 (16.2%)
Indication for CT scanning	Other/mixed	141 (26.6%)
	Non-traumatic vascular	130 (24.5%)
	Infection or inflammation	108 (20.3%)
	Trauma	90 (16.9%)
	Acute bowel pathology	35 (6.6%)
	Urolithiasis	16 (3.0%)
	Acute oncology	11 (2.1%)
Use of intravenous CT contrast agents	Multiple phases	200 (37.7%)
	Venous phase	197 (37.1%)
	Arterial phase	97 (18.3%)
	Unenhanced	37 (7.0%)
Use of gastrointestinal CT contrast agents	No oral or rectal contrast agents	339 (63.8%)
	Oral contrast agents only	136 (25.6%)
	Both oral and rectal contrast agents	43 (8.1%)
	Rectal contrast agents only	13 (24.5%)
Time of CT scanning	Before midnight	404 (76.1%)
	After midnight	127 (23.9%)

^a^Median with interquartile range between parentheses

### Clinical reasoning quality and number of CT scans: temporal changes

**Fig. 2:**
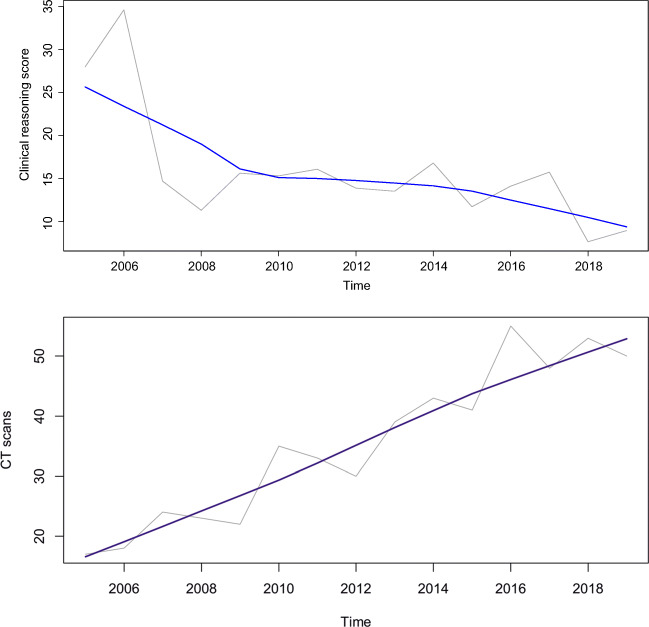
*Upper panel*: clinical reasoning scores per year (gray line), calculated as (*a* / (*b* × *c*)) × 100%, where *a* denotes the number of differential diagnoses on the CT request forms that matched the CT diagnoses, *b* denotes the total number of differential diagnoses on the CT request forms, and *c* denotes the number of corresponding CT scans performed in each year. Therefore, it should be emphasized that the clinical reasoning scores along the y-axis are given as percentages. The blue line represents the non-parametric locally estimated scatterplot smoothing (LOESS) fit (Mann-Kendall tau of −0.829, *p* < 0.001). *Lower panel*: number of abdominal CT scans during on-call hours on 36 randomly sampled unique calendar days per year (gray line), with LOESS fit in blue (Mann-Kendall tau of 0.790, *p* < 0.001)

The median annual clinical reasoning score was 14.7% (IQR: 12.2 to 16.0%; range: 7.7 to 34.6%). Clinical reasoning scores significantly decreased over time (Mann-Kendall tau of −0.390, *p* < 0.048) (Fig. 2). The median annual number of abdominal CT scans during on-call hours on the 36 randomly sampled unique calendar days was 35 (IQR: 23.25 to 46.75; range: 17– 55). The number of abdominal CT scans significantly increased over time (Mann-Kendall tau of 0.790, *p* < 0.001) (Fig. 2).

### Clinical reasoning skills: determinants

There was a significant association between the quality of clinical reasoning and patient age (*β* coefficient of 0.210, *p* = 0.002). The quality of clinical reasoning was not significantly associated with patient gender, requesting department, or time of CT scanning (Table 3).

**Table 3:** Linear regression analysis on the association of the quality of clinical reasoning with patient age, patient gender, requesting department, and time of CT scanning

Variable	Category	*β* coefficient	95% CI	*p*-value
Patient age	Continuous variable	0.210	0.081 to 0.340	0.002
Patient gender^a^	Female	−2.528	−7.262 to 2.205	0.295
Requesting department^b^	Internal medicine	−0.431	−7.124 to 6.262	0.899
	Emergency medicine	−3.233	−10.480 to 4.014	0.381
	Intensive care	−1.943	−13.986 to 10.100	0.751
	Other	−2.901	−9.473 to 3.671	0.386
Time of CT scanning^c^	After midnight	2.989	−2.407 to 8.386	0.277

*CI* confidence interval

^a^Male gender was used as reference category

^b^Surgery was used as reference category

^c^Before midnight was used as reference category

## Discussion

Our study has four main findings. First, in patients with suspected acute abdominal pathology who are scheduled to undergo CT scanning during on-call hours, referring physicians generally have difficulties in making an accurate pretest (differential) diagnosis. Second, clinical reasoning quality of physicians who request abdominal CT scans in these patients has significantly deteriorated over the years. Third, the number of CT scans has shown a significant increase during the same time frame. Fourth, clinical reasoning quality was significantly lower in younger patients in this setting.

Our results underline the importance of CT scanning complementary to clinical history, physical examination, and laboratory findings, in increasing or decreasing the likelihood of acute diseases in the abdomen in the appropriate clinical setting. Most importantly, they confirm our hypothesis that the clinical reasoning quality of physicians who request acute abdominal CT scans has deteriorated over the years, while the number of CT scans has shown an opposite temporal trend. We speculate that this is mainly due to the CT scan capacity that increased over the years. In 2005, our hospital had two CT systems, which increased to three, four, and five CT systems in the course of 2006, 2008, and 2015, respectively. Notably, the largest relative increase in CT scan capacity occurred in the course of 2006 (150%), which coincided with the largest subsequent drop in clinical reasoning quality (Fig. 2). Because of the virtually unlimited CT scan capacity, the need to carefully select those patients who would benefit most from CT scanning has been eliminated. We believe this has contributed to a culture change in which medical doctors spend less efforts to and have become less skilled at clinical reasoning, in essence exchanging clinical reasoning quality for CT scan quantity. Perhaps an increase in clinical productivity and other tasks (e.g., administrative) of referring physicians has also resulted in worse clinical reasoning, although there are no data available to support this notion. It remains unclear why clinical reasoning was worse in younger patients. Whether or not this is related to younger generations demanding more imaging, defensive medicine practices in younger patients, or other reasons, requires further investigation.

Our study may have some implications. On one hand, attempts may be made to improve clinical reasoning of physicians who request CT scans. Education has been suggested as the most effective approach to reach this goal, and radiologists have been urged to accept this educational responsibility [3]. This may be attempted by participating in undergraduate medical education and by providing feedback to referring physicians (e.g., on an individual basis or during multidisciplinary meetings — highlighting the importance of communication with referring physicians), all of which take place at our institution. On the other hand, if poor clinical reasoning of referring physicians will be accepted as the status quo, healthcare policy makers may consider assigning more value to the role of medical imaging and interpreting radiologists. This may be realized by allocating more funds to train and hire radiology staff, because they save healthcare costs elsewhere, namely referring physician’s efforts and time spent on comprehensive clinical history taking, physical examination, requesting and interpreting laboratory findings, and establishing an accurate differential diagnosis. In light of ongoing technical developments in cross-sectional imaging that further decrease acquisition time (CT, MRI and PET) or decrease radiation dose (CT and PET) [11–14], it is not inconceivable that CT and other cross-sectional imaging modalities will take over more and more upfront diagnostic tasks from referring physicians.

Experts in the field of abdominal imaging have previously reported that many patients with acute abdominal pain are referred for imaging without a clear pretest diagnosis, and that in only a small proportion of patients a confident and accurate diagnosis can be made solely on the basis of medical history, physical examination, and laboratory test findings [8]. These expert statements are completely in line with the findings of the present study. Previous studies have also shown that the use of cross-sectional imaging (particularly CT and MRI in adults) keeps on rising, including abdominal CT utilization during on-call hours [1, 2]. These findings are again no different from that of the present study. Most importantly, however, studies investigating (temporal changes in) clinical reasoning quality of physicians who request cross-sectional imaging have been lacking so far.

The present study had some limitations. First, this study was performed in a tertiary care center in a Western European country without any CT scan capacity issues. The results may be different in other hospitals with different patient populations and CT scan capacities. It should also be noted that 61.6% of patients in this study were male, for which we do not have a clear explanation. Second, the results only apply to acute abdominal CT scans performed during on-call hours. Further research is necessary to investigate if our findings are also applicable to other clinical settings in which cross-sectional imaging is requested. Third, it can be argued that the CT request forms may not have contained all clinical reasoning considerations that may have been communicated by phone. However, providing adequate clinical information with a differential diagnosis on the CT request form can be considered a requisite to avoid miscommunication and errors, and in our experience the telephonic and written communications match in this setting. Fourth, annual data on the number of patient consultations related to acute abdominal pathology, patient expectations, clinical status and gravity of their clinical presentation, staffing of the different departments in our hospital, training level of the persons who filled in the CT request forms (e.g., medical specialist vs. non-medical specialist, and years of experience), allegations against medical doctors, and defense medicine practices, were not available, as a result of which it was not possible to correlate these metrics to clinical reasoning quality. These topics should be investigated by further studies. It also remains unclear if new guidelines have an effect on clinical reasoning quality. Nevertheless, after a national guideline on diagnostic tests for acute abdominal pain in adults (excluding abdominal pain after trauma, known intra-uterine pregnancy, gastro-intestinal hemorrhage, hemorrhagic shock, chronic conditions, recurrent abdominal pain, and abdominal pain within 30 days after abdominal surgery) was published in 2013 (which recommends ultrasonography as first-line imaging, followed by CT if ultrasonography is negative or inconclusive, unless the patient is critically ill) [15], there was no clear trend change with regard to clinical reasoning quality. Fifth, CT scans were not evaluated in terms of appropriateness, influence on patient management, or effect on patient outcome. This was beyond the scope of the present study. Previous work has already shown that CT results in emergency department settings frequently change physicians’ diagnoses and admission decisions [16].

In conclusion, the clinical reasoning quality of physicians who request abdominal CT scans during on-call hours has deteriorated over time. Clinical reasoning appears to be worse in younger patients.

## Abbreviations

CI: Confidence interval
IQR: Interquartile range
LOESS: Locally estimated scatterplot smoothing

## Declarations

### Guarantor

The scientific guarantor of this publication is Thomas C. Kwee.

### Statistics and biometry

No complex statistical methods were necessary for this paper.

### Study subjects or cohorts overlap

The cases that were selected for the present study were derived from a previously published study that had a different purpose, namely to investigate temporal changes in the utilization and patient impact of abdominal CT during duty shifts in terms of negative scan proportion, same-day hospital discharge, and incidental findings. (Mengou IV, Yakar D, Kasalak Ö, Kwee TC (2021) Towards a benchmark of abdominal CT use during duty shifts: 15-year sample from the Netherlands. Abdom Radiol (NY) 46: 1761-1767).

### Methodology

• retrospective

• observational

• performed at one institution
