# Correction: Did medical doctors who order abdominal CT scans during on-call hours truly become worse at clinical reasoning? Yes, they did

**DOI:** 10.1007/s00330-023-09821-8

**Published:** 2023-06-20

**Authors:** Selin Ersoydan, Derya Yakar, Ömer Kasalak, Thomas C. Kwee

**Affiliations:** Department of Radiology, University Medical Center Groningen, University of Groningen, Groningen, the Netherlands

**Correction: European Radiology (2022) 33:1015-1021**

**https://doi.org/10.1007/s00330-022-09121-7**

The original version of this article, published on 7 September 2022, unfortunately contained some mistakes.

The authors noted an error in the calculation of the annual clinical reasoning scores. The original legend with the explanation of this calculation and the display of the upper panel of Fig. 2 were incorrect, and have been replaced with a corrected version. Some sentences in the results of the abstract and main text have also been corrected based on this recalculation (see below). However, these corrections do not change the interpretation of the results and the conclusions we have drawn.

Abstract, results section, corrected first two sentences:

The median annual clinical reasoning score was 14.7% (interquartile range: 12.2 to 16.0%; range: 7.7 to 34.6%). The quality of clinical reasoning significantly decreased between 2005 and 2019 (Mann–Kendall Tau of − 0.390, *p* = 0.048), while the number of abdominal CT scans significantly increased (Mann–Kendall tau of 0.790, *p* < 0.001).

Main text, results section, corrected paragraph “Clinical reasoning quality and number of CT scans: temporal changes”:

The median annual clinical reasoning score was 14.7% (IQR: 12.2 to 16.0%; range: 7.7 to 34.6%). Clinical reasoning scores significantly decreased over time (Mann–Kendall tau of − 0.390, *p* < 0.048) (Fig. 2). The median annual number of abdominal CT scans during on-call hours on the 36 randomly sampled unique calendar days was 35 (IQR: 23.25 to 46.75; range: 17–55). The number of abdominal CT scans significantly increased over time (Mann–Kendall tau of 0.790, *p* < 0.001) (Fig. 2).

Figure 2, Upper panel

Clinical reasoning scores per year (gray line), calculated as (a/b) × 100%, where a denotes the number of differential diagnoses on the CT request forms that matched the CT diagnoses and b denotes the total number of differential diagnoses on the CT request forms, for each year. The blue line represents the non-parametric locally estimated scatterplot smoothing (LOESS) fit (Mann–Kendall Tau of − 0.390, *p* = 0.048).

The upper panel of Fig. 2 has also been replaced with a newer version.
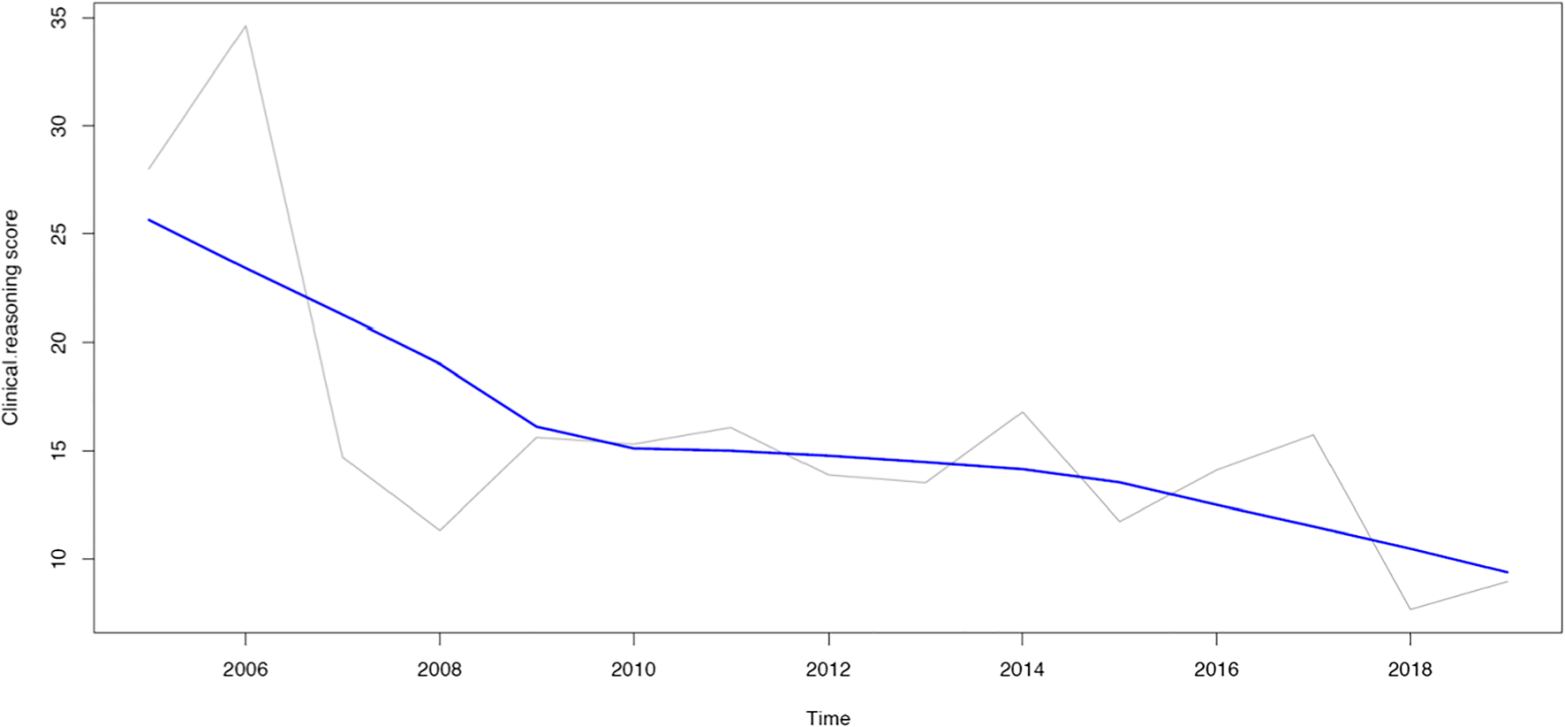


The original article has been corrected.

